# Direct Vapor Growth of 2D Vertical Heterostructures with Tunable Band Alignments and Interfacial Charge Transfer Behaviors

**DOI:** 10.1002/advs.201802204

**Published:** 2019-02-14

**Authors:** Weihao Zheng, Biyuan Zheng, Changlin Yan, Ying Liu, Xingxia Sun, Zhaoyang Qi, Tiefeng Yang, Ying Jiang, Wei Huang, Peng Fan, Feng Jiang, Wei Ji, Xiao Wang, Anlian Pan

**Affiliations:** ^1^ Key Laboratory for Micro–Nano Physics and Technology of Hunan Province State Key Laboratory of Chemo/Biosensing and Chemometrics and College of Materials Science and Engineering Hunan University Changsha Hunan 410012 China; ^2^ Beijing Key Laboratory of Optoelectronic Functional Material & Micro–Nano Devices Department of Physics Renmin University of China Beijing 100872 China; ^3^ School of Physics and Electronics Hunan University Changsha Hunan 410012 China

**Keywords:** band alignments, charge transfers, photoluminescence enhancement, photoluminescence quenching, van der Waals heterostructures

## Abstract

2D vertical van der Waals (vdW) heterostructures with atomically sharp interfaces have attracted tremendous interest in 2D photonic and optoelectronic applications. Band alignment engineering in 2D heterostructures provides a perfect platform for tailoring interfacial charge transfer behaviors, from which desired optical and optoelectronic features can be realized. Here, by developing a two‐step chemical vapor deposition strategy, direct vapor growth of monolayer PbI_2_ on monolayer transition metal dichalcogenides (TMDCs) (WS_2_, WSe_2_, or alloying WS_2(1−_ *_x_*_)_Se_2_ *_x_*), forming bilayer vertical heterostructures, is demonstrated. Based on the calculated electron band structures, the interfacial band alignments of the obtained heterostructures can be gradually tuned from type‐I (PbI_2_/WS_2_) to type‐II (PbI_2_/WSe_2_). Steady‐state photoluminescence (PL) and time‐resolved PL measurements reveal that the PL emissions from the bottom TMDC layers can be modulated from apparently enhanced (for WS_2_) to greatly quenched (for WSe_2_) compared to their monolayer counterparts, which can be attributed to the band alignment–induced distinct interfacial charge transfer behaviors. The band alignment nature of the heterostructures is further demonstrated by the PL excitation spectroscopy and interlayer exciton investigation. The realization of 2D vertical heterostructures with tunable band alignments will provide a new material platform for designing and constructing multifunctional optoelectronic devices.

Energy band alignment engineering at the interface of semiconductor heterostructure is crucial for tuning its optical and electronic properties and realizing new functional photonic and optoelectronic devices.^1^, ^2^, ^3^, ^4^, ^5^, ^6^, ^7^, ^8^ Compared to conventional semiconductor heterostructures, the emerging 2D layered heterostructures with van der Waals (vdW) interaction between the layers offer a perfect platform for band alignment engineering via stacking different layers with distinct band structures.^9^, ^10^ In addition, the formed atomically sharp interfaces in these heterostructures facilitate many interfacial photophysics processes, such as interfacial charge transfer,^11^, ^12^, ^13^, ^14^ which can be utilized for high‐performance device applications. For 2D layered heterostructures with a type‐II (staggered) band alignment, photogenerated electrons or holes can transfer across the interface and be separated at different layers due to the band offset, making these heterostructures ideal for light harvesting and photodetection.^15^, ^16^, ^17^ In contrast, with a type‐I (straddling) band alignment, the photogenerated electrons and holes in the wider bandgap layer can efficiently transfer into the layer with a narrower bandgap, leading to an increased carrier population and enhanced photoluminescence emission, which has the potential for light‐emitting applications.^18^, ^19^, ^20^

Monolayer transition metal dichalcogenides (TMDCs) with direct bandgap covering a wide energy range are ideal candidates for preparing the 2D layered heterostructures.^21^, ^22^, ^23^ However, due to the band structures, it is difficult to form type‐I band alignment from typical TMDCs. Although the band alignment of 2D layered heterostructure can be influenced by introducing an external electric field or mechanical field,^24^, ^25^ the small tunability and poor controllability largely prevent these methods from practical applications. In contrast, by carefully selecting the layered materials, the engineering of band structure configurations and even the tuning of the type of the band alignments, e.g., the transition from type‐II to type‐I, in heterostructures can be realized. To this end, monolayer lead iodide (PbI_2_) with a wider bandgap and higher light absorption coefficient^26^, ^27^, ^28^ provides the possibility of forming vdW heterostructures with monolayer TMDCs for different band alignments. Furthermore, the 2D vdW heterostructure with a large‐range tunable band alignment can be obtained by introducing the alloying TMDCs.^29^, ^30^, ^31^, ^32^, ^33^, ^34^

Here, we demonstrate, for the first time, a direct vapor phase growth of vdW heterostructures composed of monolayer PbI_2_ and monolayer alloying WS_2(1−_ *_x_*_)_Se_2_ *_x_* (0 ≤ *x* ≤ 1) through a two‐step chemical vapor deposition (CVD) strategy. By tuning different *x* values in the bottom alloying TMDC monolayers, the band alignment of these heterostructures emerges a transition from type‐I (PbI_2_/WS_2_, *x* = 0) to type‐II (PbI_2_/WSe_2,_ *x* = 1), with a transition point at *x* = 0.67 (PbI_2_/WS_0.67_Se_1.33_). Meanwhile, the photoluminescence (PL) emission from the bottom TMDC layers can also be modulated from apparently enhanced (for WS_2_) to greatly quenched (for WSe_2_) compared to their pristine monolayer counterparts, which can be attributed to the band alignment–induced distinct interfacial charge transfer behaviors. We further demonstrate the type‐I and type‐II band alignment nature of the PbI_2_/WS_2_ and PbI_2_/WSe_2_ heterostructures by photoluminescence excitation (PLE) spectroscopy and the study of interlayer exciton (I‐exciton) behaviors, respectively. These 2D layered heterostructures with engineered band alignment not only provide a platform for fundamental physical investigations but also have potential applications in integrated electronic and optoelectronic applications.

PbI_2_/TMDC vertical heterostructures were synthesized via a two‐step CVD strategy (**Figure** **1**a). At first, the bottom TMDC (WS_2_, WSe_2_, or the alloying WS_2(1−_ *_x_*_)_Se_2_ *_x_*) monolayers were grown with the same method described in our previous works.^29^, ^33^, ^34^ Briefly, a quartz boat loaded with the WS_2_, WSe_2_, or the mixed powders was placed at the heating zone while a substrate (Si/300 nm SiO_2_) was placed at the downstream region of a tube furnace. The furnace was then stably heated to 1050 °C within 40 min and maintained at this temperature for 5 min, with the Ar gas flow at a rate of 50 sccm for transporting the source to the substrate. The alloying WS_2(1−_ *_x_*_)_Se_2_ *_x_* monolayers with the desired *x* value can be obtained by adjusting the mass ratio between the WS_2_ and WSe_2_ powders (see Figure S1 in the Supporting Information). In the second step, the substrate with as‐grown TMDC monolayers was rapidly transferred to the downstream of the other furnace with a quartz boat loaded with PbI_2_ powder placed at the heating zone. The furnace was then heated to 400 °C within 30 min and maintained at this temperature for 20 min with the Ar gas flow at a rate of 30 sccm. During the growth, PbI_2_ prefers to crystallize on the as‐grown TMDCs to form heterostructures instead of forming monolayers on the substrate, probably due to a higher absorption energy of TMDCs compared to the that with the substrate.^35^ By varying the deposition position (relating to the deposition temperature) of the substrate in the second step, the thickness of PbI_2_ can be controlled in general and the heterostructures comprising of monolayer WS_2_ and monolayer PbI_2_ were obtained. Since the PbI_2_ layers were grown at such a low temperature, the bottom TMDC monolayers were prevented from thermal damage during the formation of heterostructures.

**Figure 1:**
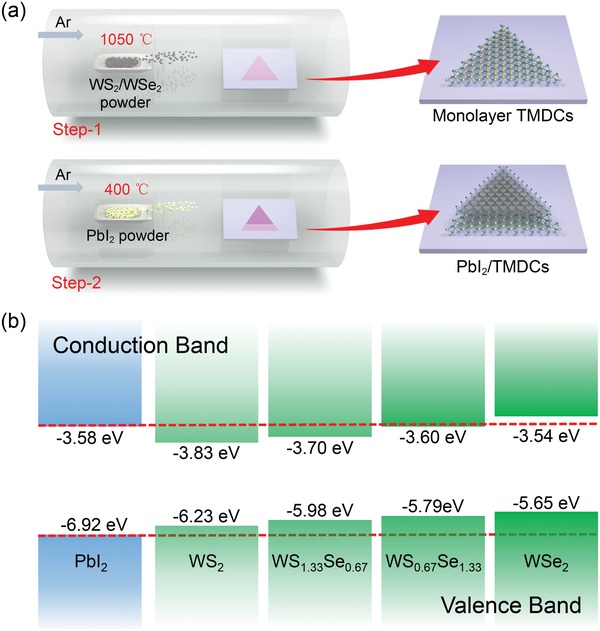
a) Illustration of the two‐step CVD strategy for the growth of PbI_2_/TMDC heterostructure. TMDCs (WS_2_, WSe_2_, or alloying WS_2(1−_ *_x_*_)_Se_2_ *_x_*). b) Calculated energy band structures of monolayer PbI_2_, monolayer WS_2(1−_ *_x_*_)_Se_2_ *_x_* (*x* = 0, 0.33, 0.67, and 1). Red dash lines indicate the CBM and VBM of PbI_2_. These band structures suggest a possible band alignment transition from type‐I (PbI_2_/WS_2_) to type‐II (PbI_2_/WSe_2_).

On the basis of ab initio simulation calculations (HSE06) of the electronic band structure of WS_2_, WSe_2_, alloying WS_2(1−_ *_x_*_)_Se_2_ *_x_* and PbI_2_, the band alignments of PbI_2_/WS_2_ and PbI_2_/WSe_2_ show straddling and staggered configurations (Figure 1b; see the “Experimental Section” for detail), respectively. Thus, type‐I and type‐II vdW heterostructures with distinct optical and electronic properties can be realized by constructing monolayer PbI_2_ with monolayer WS_2_ and WSe_2_, respectively. In addition, for the WS_2(1−_ *_x_*_)_Se_2_ *_x_*, with the *x* value varying from 0 to 1, both the conduction band minimum (CBM) and the valence band maximum (VBM) would become higher while the bandgap is smaller, which agrees with the previous reports.^33^, ^36^ Therefore, the band alignment of the heterostructures can be dynamically engineered from type‐I to type‐II by controlling the *x* value of the bottom WS_2(1−_ *_x_*_)_Se_2_ *_x_* monolayer and a transition point can be found with a certain *x* value at which the CBM of WS_2(1−_ *_x_*_)_Se_2_ *_x_* is equal to that of PbI_2_. This indicates that the charge transfer behaviors at the interface can be largely controlled by tuning the sign and amplitude of the band offset between the PbI_2_ and TMDCs in heterostructures.

To reveal the growth process of the top PbI_2_ monolayer on the bottom TMDCs, we took the PbI_2_/WS_2_ heterostructure as an example and recorded optical images of the heterostructures obtained with typical growth time varying from 0 to 20 min (**Figure** **2**a). The PbI_2_ was found to grow initially at the edge of WS_2_, where there are probably more dangling bonds and defects.^37^ Then, the PbI_2_ epitaxially grows across the WS_2_ surface, forming the PbI_2_/WS_2_ heterostructure. Given a long growth time, a whole covering of the top PbI_2_ on WS_2_ can be achieved. Figure 2b shows the atomic force microscope (AFM) images of typical areas marked with rectangles in an optical image of the 10 min growth sample (Figure 2a). AFM line profiles shown as insets in Figure 2b clearly demonstrate the formation of monolayer–monolayer heterostructure, with the height of uncovered WS_2_ being 0.7 nm and the heterostructure being 1.4 nm (PbI_2_ layer 0.7 nm).^38^, ^39^ High‐resolution transmission electron microscope (HRTEM) image of the heterostructure is shown in Figure 2c. Distinct Moiré patterns caused by the overlapping lattices of the PbI_2_ and WS_2_ were observed, demonstrating the formation of PbI_2_/WS_2_ vertical heterostructure as well. Fast Fourier transform (FFT) of this HRTEM image (Figure 2d) shows two sets of hexagonally arranged diffraction patterns, which can be assigned the [100] plane of PbI_2_ (yellow circles, 0.38 nm lattice spacing) and WS_2_ (red circles, 0.27 nm lattice spacing),^33^, ^39^ respectively. This large lattice mismatch (40.7%) between the PbI_2_ and WS_2_ indicates the ultrahigh tolerance of lattice mismatch in vdW heterostructure. Additional characterization of PbI_2_/WS_2_ heterostructure is provided by cross‐sectional TEM (Figure 2e), which directly gives the evidence of the stacking of the PbI_2_ above WS_2_. Meanwhile, corresponding elemental energy dispersive X‐ray spectroscopy (EDS) results of the cross section also confirm the existence of PbI_2_ and WS_2_ in the heterostructure (see Figure S2 in the Supporting Information).

**Figure 2:**
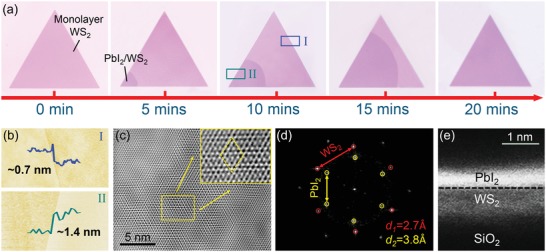
a) Optical images of the PbI_2_/WS_2_ obtained at different growth time. b) AFM images obtained at areas I (blue rectangle) and II (green rectangle) of the 10 min growth sample shown in (a). c) HRTEM image taken from the heterostructure, indicating the vertically stacked PbI_2_ and WS_2_ layers with the Moiré pattern. Scale bar: 5 nm. Inset: amplifying image from the yellow rectangle and the yellow dash rhombus indicates the periodicity of the Moiré pattern. d) FFT of the HRTEM image in panel (c). The hexagonally arranged diffraction patterns of PbI_2_ and WS_2_ are shown with yellow and red circles, respectively. The lattice spaces are marked. e) Bright‐field TEM image of the cross‐sectional morphology of PbI_2_/WS_2_ heterostructure. Scale bar: 1 nm.

In order to demonstrate the band alignment and the charge transfer behaviors, we choose three typical heterostructures, PbI_2_/WS_2_, PbI_2_/WS_0.67_Se_1.33_, and PbI_2_/WSe_2_. The optical images of these heterostructures with half‐covered PbI_2_ are shown in **Figure** **3**a–c. We observed clear optical contrast between these heterostructures and the bottom monolayers. Typical height profiles recorded from the AFM measurements are shown as insets in Figure 3a–c, indicating the formation of monolayer–monolayer heterostructures. Micro‐Raman spectroscopy was used to further characterize the heterostructures. For all spectra recorded at heterostructures, the vibrational modes of PbI_2_, containing E_g_ (75.3 cm^−1^), A_1g_ (95.5 cm^−1^), and A_2u_ (113.1 cm^−1^), were observed with relative weak intensity (Figure 3d–f), which agrees with the literature.^28^ Typical Raman peaks of A_1g(S–W)_ (417.9 cm^−1^) and E_2g_ (351.9 cm^−1^) from WS_2_ and A_1g(Se–W)_ (250.0 cm^−1^) from WSe_2_ were observed in PbI_2_/WS_2_ and PbI_2_/WSe_2_ heterostructures. The Raman spectrum of PbI_2_/WS_0.67_Se_1.33_ heterostructure shows additional vibrational modes of E_2g(S–W)_ − LA_(S–W)_ + A_1g(Se–W)_ − LA_(Se–W)_ (a mode) and A_1g(S–W–Se)_ (b mode) compared to WS_2_ and WSe_2_, demonstrating the formation of alloying layer. We noted that the Raman vibrational modes of TMDCs in heterostructures all show slight blueshifts compared to the monolayer counterparts, which is probably due to the strain effect induced by the top PbI_2_ layer.

**Figure 3:**
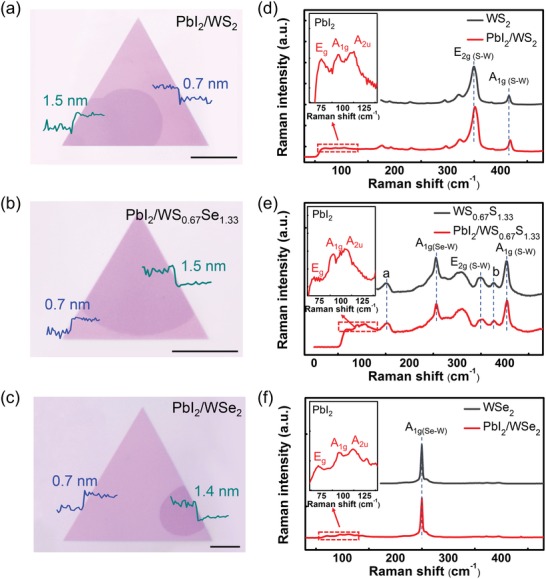
a–c) Optical images and d–f) Raman spectra of PbI_2_/WS_2_, PbI_2_/WS_0.67_Se_1.33_, and PbI_2_/WSe_2_ heterostructures. The Raman vibrational modes are labeled in the spectra. The peak *a* and peak *b* in panel (e) represent the E_2g(S–W)_ − LA_(S–W)_ + A_1g(Se–W)_ − LA_(Se–W)_ and A_1g(S–W–Se)_ modes, respectively. Dash lines are used to guide the peak positions.

Steady‐state PL spectroscopy, PL mapping, and the time‐resolved photoluminescence (TRPL) spectroscopy were performed to study the PL emission intensity and dynamics of the bottom TMDC layer in these heterostructures, which can reflect the band alignment–induced interfacial charge transfer behaviors at the interface. The PL intensity mapping (at the emission wavelength of the bottom TMDCs) of PbI_2_/WS_2_, PbI_2_/WS_0.67_Se_1.33_, and PbI_2_/WSe_2_ heterostructures is shown in **Figure** **4**a_1_–a_3_, respectively. Typical PL spectra collected in the heterostructures and the bare monolayers are compared in Figure 4b_1_–b_3_. We observed that the PL intensity of WS_2_ is significantly enhanced in PbI_2_/WS_2_ heterostructure compared to that in monolayer WS_2_. In contrast, the WSe_2_ PL in the PbI_2_/WSe_2_ heterostructure shows strong intensity quenching. Only a slight enhancement of the WS_0.67_Se_1.33_ PL was observed in the PbI_2_/WS_0.67_Se_1.33_ heterostructure. The PL enhancing or quenching of TMDCs in their corresponding heterostructures can be quantified by defining an enhancing factor of α = *I*_H_/*I*_M_, where *I*_H_ and *I*_M_ are the PL intensity of TMDCs in heterostructure and monolayer, respectively. The α values of ≈2.02, 1.12, and 0.09 were obtained in PbI_2_/WS_2_, PbI_2_/WS_0.67_Se_1.33_, and PbI_2_/WSe_2_, respectively, clearly demonstrating the transition from PL enhancement to PL quenching. In addition, we noted that the PL of the bottom monolayer all shows an obvious redshifted peak (≈32 meV in PbI_2_/WS_2_, ≈22 meV in PbI_2_/WS_0.67_Se_1.33_, and ≈21 meV in PbI_2_/WSe_2_) in the heterostructure, which suggests a strong interlayer coupling between the PbI_2_ and bottom layers.^40^ TRPL spectroscopy was performed to study the PL dynamics in these heterostructures (Figure 4c_1_–c_3_). For both PbI_2_/WS_2_ and PbI_2_/WS_0.67_Se_1.33_, the PL decay curves of the TMDCs in monolayer and heterostructures are quite similar, with only a slight shift to longer time in PbI_2_/WS_2_. In contrast, the lifetime of WSe_2_ PL is dramatically decreased in PbI_2_/WSe_2_ heterostructure compared to the monolayer, implying an additional carrier decay channel of the WSe_2_ being created in the heterostructure.

**Figure 4:**
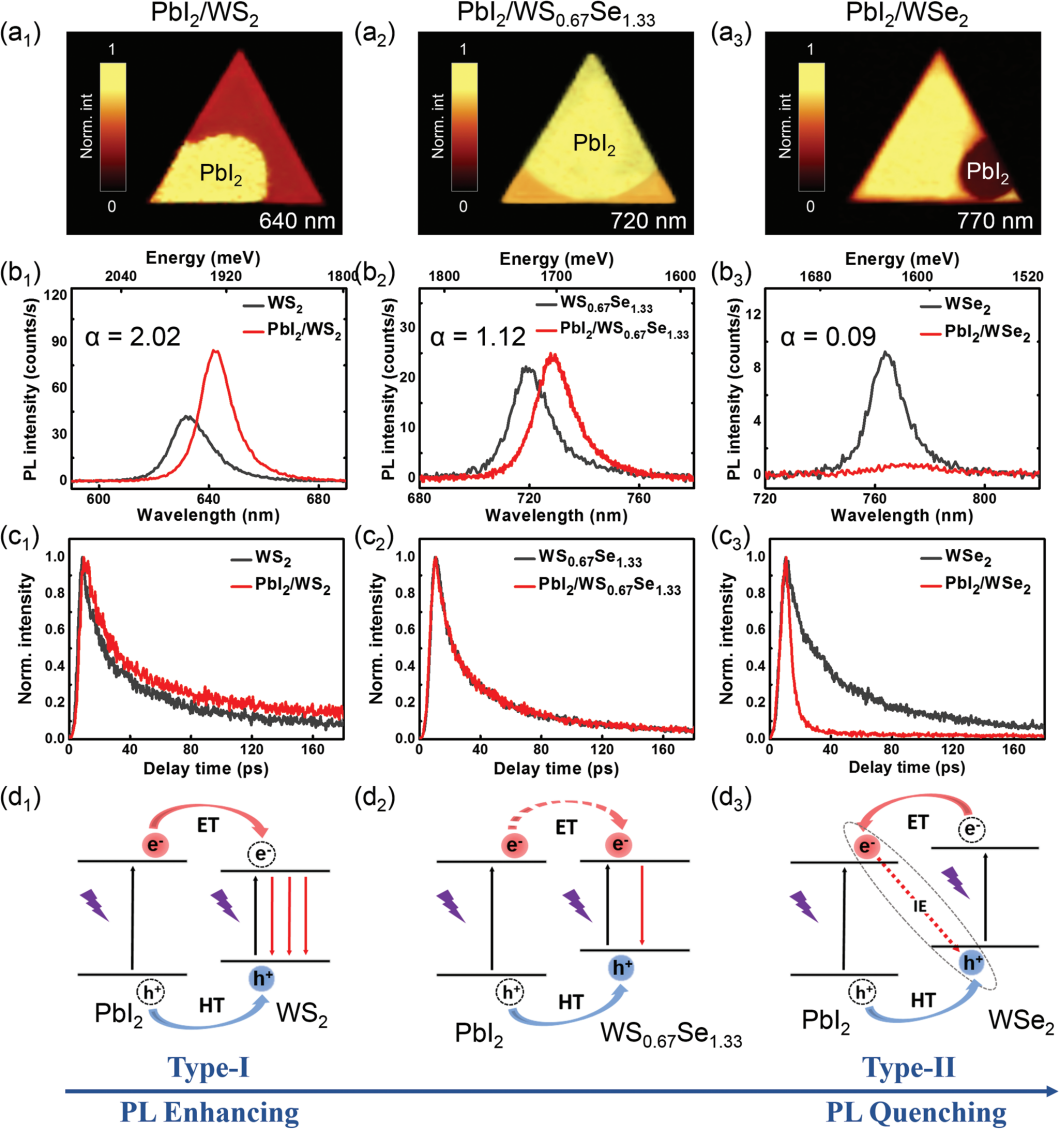
a_1_–a_3_) 2D PL mapping, b_1_–b_3_) typical PL spectra, and c_1_–c_3_) TRPL of TMDCs in PbI_2_/WS_2_, PbI_2_/WS_0.67_Se_1.33_, and PbI_2_/WSe_2_ heterostructures. The PbI_2_‐covered regions are marked. Values of enhancing factor α are marked. d_1_–d_3_) Schematic of the charge transfer processes in PbI_2_/WS_2_, PbI_2_/WS_0.67_Se_1.33_, and PbI_2_/WSe_2_ heterostructures. The black straight arrow represents the excitation while the red straight arrow represents the direct recombination. The red dash arrow represents the indirect interlayer recombination. ET: electron transfer, HT: hole transfer.

The observed different PL behaviors in different heterostructures can be well interpreted by the band alignment–induced charge transfer processes. For PbI_2_/WS_2_ heterostructure with type‐I band alignment, upon laser excitation, both the hole and electron can transfer from the PbI_2_ to the WS_2_ layer, leading to an increased carrier population and thus enhanced WS_2_ PL. In addition, the transfer time between the PbI_2_ and WS_2_ layer should be at very short time scale, and these transferred carriers should recombine through the same channel of the carriers which generated in WS_2_ layer, resulting in the mostly unchanged PL decay curve. For PbI_2_/WS_0.67_Se_1.33_ heterostructure, the CBM of WS_0.67_Se_1.33_ is roughly equal to that of PbI_2_ monolayer, which leads to the suppression of the electron transfer from PbI_2_ to WS_2_. Therefore, we did not observe a large enhancement of the PL like in the PbI_2_/WS_2_ heterostructure. For PbI_2_/WSe_2_ heterostructure with type‐II band alignment, the photogenerated electron and hole are, respectively, separated into PbI_2_ and WSe_2_ layers after charge transfer, leading to the extra decay channel for the electrons and thereby resulting the quenching of the WSe_2_ PL.

We further performed PLE experiments to experimentally demonstrate the type‐I band alignment nature of PbI_2_/WS_2_ heterostructure. The PL enhancement factor α as a function of excitation laser wavelength is shown in **Figure** **5**a. A rapid decrease of α value from ≈2 to ≈1 was observed in the excitation wavelength range of 510–550 nm, which matches the absorption edge of PbI_2_ as reported in the previous work.^28^ Within the range of 400–510 nm, both the PbI_2_ and WS_2_ layers were excited, where photogenerated carriers in PbI_2_ can be injected into the WS_2_. However, for excitation wavelength longer than 550 nm, only the WS_2_ layer was excited without external carrier injection so that the WS_2_ PL intensity in heterostructure equals to that of the monolayer WS_2_. The PLE experiment corroborated that the PL enhancement of WS_2_ originates from the charge transfer of PbI_2_, demonstrating the type‐I band alignment of PbI_2_/WS_2_ heterostructure. For PbI_2_/WSe_2_ heterostructure with type‐II alignment, the I‐excitons were observed and characterized. The PL spectrum collected from the PbI_2_/WSe_2_ heterostructure with a long integration time is depicted in Figure 5b. The whole spectral profile can be fitted by two Lorentzian peaks, the positions of which are located at 774.4 and 800.4 nm, respectively. We attribute the 774.4 and 800.4 nm peaks to the A‐exciton of WSe_2_ and I‐exciton, respectively. The experimentally observed energy difference between the A‐exciton and I‐exciton is 52 meV, which agrees with the energy difference between the CBM of WSe_2_ and PbI_2_ obtained from theoretical calculation (Figure 1b). We also performed TRPL measurement on the I‐exciton and observed an additional ultralong lifetime of 642 ps at 800.4 nm, which is consistent with the indirect nature of the I‐exciton (Figure 5c).^41^

**Figure 5:**
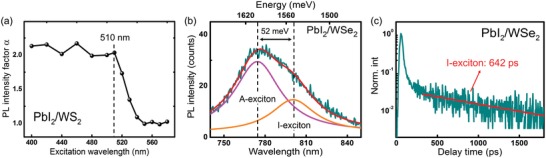
a) PL enhancement factor α as a function of excitation laser wavelength. b) PL spectrum collected from the PbI_2_/WSe_2_ heterostructure. c) TRPL curve of the I‐exciton at 800.4 nm showing an additional ultralong lifetime with a value of 642 ps. The fast decay originates from the A‐excitons which are spectrally overlapped at this wavelength.

In summary, we have theoretically and experimentally demonstrated the band alignment engineering of direct vapor growth 2D vdW heterostructures, which consist of monolayer PbI_2_ and monolayer TMDCs such as WS_2_, WSe_2_, and the alloying WS_2(1−_ *_x_*_)_Se_2_ *_x_*. These heterostructures were characterized by AFM, TEM, Raman spectroscopy, steady‐state spectroscopy, and TRPL spectroscopy. The obtained PbI_2_/WS_2_ heterostructure has the type‐I band alignment with enhanced WS_2_ PL emission compared to the monolayer, which agrees well with the charge transfer behavior in type‐I heterostructure. In contrast, PbI_2_/WSe_2_ heterostructure with the type‐II band alignment shows quenched WSe_2_ PL and much shorter PL lifetime, which is due to the band alignment–induced extra carrier decay channel and the separation of electrons and holes in different layers. In addition, interlayer excitons with much longer lifetime have also been observed in these type‐II heterostructures. We have further shown that the band alignment can be precisely tuned by introducing the WS_2(1−_ *_x_*_)_Se_2_ *_x_* alloying monolayer to prepare PbI_2_/WS_2(1−_ *_x_*_)_Se_2_ *_x_* heterostructures. Our work offers important guidance for preparing 2D vdW heterostructures with desirable band alignment, potentially leading to the realization of functional 2D photonic and optoelectronic devices.

## Experimental Section

*Density Functional Theory Calculation*: Density functional (DF) theory calculations were performed using the generalized gradient approximation for the exchange‐correlation potential, the projector augmented wave method, and a plane‐wave basis set as implemented in the Vienna ab initio simulation package (VASP).^42^, ^43^ van der Waals forces were considered at the self‐consistent vdW–DF level, which includes of a nonlocal correlation functional and an exchange functional in the optB86b form (optB86b–vdW).^44^, ^45^ This functional allows the accurate description of the structural properties of layered heterostructures.^46^, ^47^, ^48^ Electronic structures were calculated using the hybrid HSE06 functionals.^49^ Kinetic energy cutoffs of 500 and 400 eV for the plane‐wave basis sets were adopted for geometric and electronic structural calculations of the unit and super cells, respectively. A unit cell was used to model WSe_2_, WS_2_, and PbI_2_ monolayers while a 3 × 3 × 1 supercell was employed to model WS_2_ *_x_*Se_2(1−_ *_x_*_)_ alloys. Two *k*‐meshes of 9 × 9 × 1 and 3 × 3 × 1 were adopted to sample the first Brillouin zones of the unit and super cells, respectively. A vacuum layer of 15 Å was used to eliminate the image interactions from adjacent unit cells along the normal layer direction. The cell shape and volume of all configurations and all atomic positions were fully relaxed until the residual force per atom was less than 0.01 eV Å^−1^. The band edges were aligned using the vacuum level.

Two ordered substitution models were used for considering the WS_0.67_Se_1.33_ and WS_1.33_Se_0.67_ alloys, as shown in Figure S3 (Supporting Information). Such an ordered substitution model substantially reduces computational costs and complicity, which was found to be able to offer good consistency with models fully considering disorders.^50^ All the S atoms and Se atoms were evenly distributed on both sides. Calculations were performed at the Physics Lab of High‐Performance Computing of Renmin University of China and the Shanghai Supercomputer Center.

*Characterizations of As‐Grown PbI_2_/TMDC Heterostructures*: AFM measurements were in situ performed with a Bruker Dimension ICON AFM with Pt/Ir‐coated tips (ACCESS EFM). For TEM characterizations, the WS_2_ monolayers were first transferred onto a grid through a polymethyl methacrylate (PMMA)‐assisted positioning transfer method. Monolayers on SiO_2_/Si wafer were coated with PMMA (950 K, A3) via spin‐coating at a speed of 2000 rpm for 1 min, and then maintained 2 h for baking the wafer at 180 °C. After immersing into the KOH (15 m) solution for 12 h, the PMMA film was taken out from the KOH solution and swilled fully with deionized water. The cleaned PMMA film was then removed onto a grid of copper, and this grid was exposed to acetone vapor at 40 °C in the atmosphere for the PMMA removing. Finally, the target WS_2_ monolayers on the grid of copper were obtained for the step‐2 growth process to form PbI_2_/WS_2_ heterostructures on grid which can be used for the TEM characterizations.

*Micro‐Raman Spectra and Steady‐State PL Spectra Measurements*: Micro‐Raman spectra were measured using a confocal microscope (WITec, alpha‐300) with a 532 nm diode continuous‐wave laser as the excitation. The laser beam was focused on the sample with a spot diameter of ≈800 nm from the top by an objective lens (50×, Zeiss, 0.75 NA), while Raman signal was collected by the same objective lens. Steady‐state PL spectra were measured using the same confocal microscope but with 405 nm continuous‐wave laser as the excitation.

*TRPL Experiments*: TRPL experiments were performed using a confocal microscope (WITec, alpha‐300) as the collect device, and the emission signal was reflected into a streak camera (C10910, Hamamatsu) by Ag mirrors. A Ti:Sapphire laser pulsed at 400 nm (repetition rate of 80 MHz, pulse width of 80 fs) as the light source. The 400 nm output was generated by an 800 nm laser from a mode‐locked oscillator (Tsunami 3941‐X1BB, Spectra‐Physics) after a barium metaborate (BBO) crystal. The laser beam was focused on to the sample with a spot diameter of ≈3 μm from the top by an objective lens (50×, Zeiss, 0.75 NA), while PL emission was collected by the same objective lens. All the spectra were collected upon the laser excitation with a fixed pump power of 9.96 × 10^−8^ J pulse^−1^ cm^−2^.

## Conflict of Interest

The authors declare no conflict of interest.

## Supporting information

Supplementary
