# Supplementary material for: Direct Vapor Growth of 2D Vertical Heterostructures with Tunable Band Alignments and Interfacial Charge Transfer Behaviors
Source: Adv Sci (Weinh). 2019 Feb 14;6(7):1802204. doi: 10.1002/advs.201802204 (PMC6446596; doi:10.1002/advs.201802204)
Supplement: Supplementary file 1 — Supplementary [file ADVS-6-1802204-s001.pdf]

Copyright WILEY-VCH Verlag GmbH & Co. KGaA, 69469 Weinheim, Germany, 2019.

Supporting Information

for *Adv. Sci.*, DOI: 10.1002/advs. 201802204

Direct Vapor Growth of 2D Vertical Heterostructures with Tunable Band Alignments and Interfacial Charge Transfer Behaviors

Weihao Zheng, Biyuan Zheng, Changlin Yan, Ying Liu, Xingxia Sun, Zhaoyang Qi, Tiefeng Yang, Ying Jiang, Wei Huang, Peng Fan, Feng Jiang, Wei Ji, Xiao Wang, and Anlian Pan**

Supporting Information

Direct Vapor Growth of Two-Dimensional Vertical Heterostructures with Tunable Band Alignments and Interfacial Charge Transfer Behaviors

Weihao Zheng[†]*, Biyuan Zheng*[†]*, Changlin Yan, Ying Liu, Xingxia Sun, Zhaoyang Qi, Tiefeng Yang, Ying Jiang, Wei Huang, Peng Fan, Feng Jiang, Wei Ji, Xiao Wang*[*]*, Anlian Pan*[*]

W. H. Zheng, B. Y. Zheng, C. L. Yan, Y. Liu, X. X. Sun, Z. Y. Qi, T. F. Yang, W. Huang, P. Fan, F. Jiang, Prof. A. L. Pan

Key Laboratory for Micro-Nano Physics and Technology of Hunan Province, State Key Laboratory of Chemo/Biosensing and Chemometrics and College of Materials Science and Engineering

Hunan University

Changsha Hunan 410012, China

Dr. Y. Jiang, Prof. X. Wang, Prof. A. L. Pan

School of Physics and Electronics
Hunan University
Changsha 410012, China

C. L. Yan, Prof. W. Ji

Beijing Key Laboratory of Optoelectronic Functional Material & Micro-Nano Devices, Department of Physics

Renmin University of China

Beijing 100872, China

Corresponding Authors *E-mail: xiao_wang@hnu.edu.cn; anlian.pan@hnu.edu.cn

[†]The author contributed equally to this work.

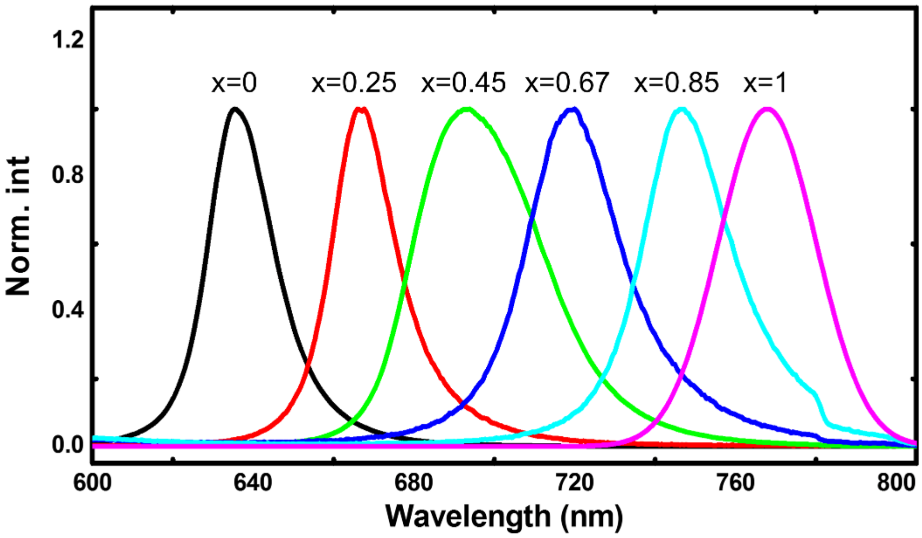

Figure S1. PL spectrum of the $WS_{2(1-x)}Se_{2x}$ monolayers with tunable composition x = 0, 0.25, 0.45, 0.67, 0.85, 1. Excited with a 532 nm diode continuous wave laser. The x value of alloying $WS_{2(1-x)}Se_{2x}$ can be determined by a ternary semiconductor alloying equation of: $\mathrm{E}_g(\mathrm{a}) = a\mathrm{E}_{gWS2} + (1-a)\mathrm{E}_{gWSe2}$, where a is the S/(S+Se) ratio and x = 1-a.[1]

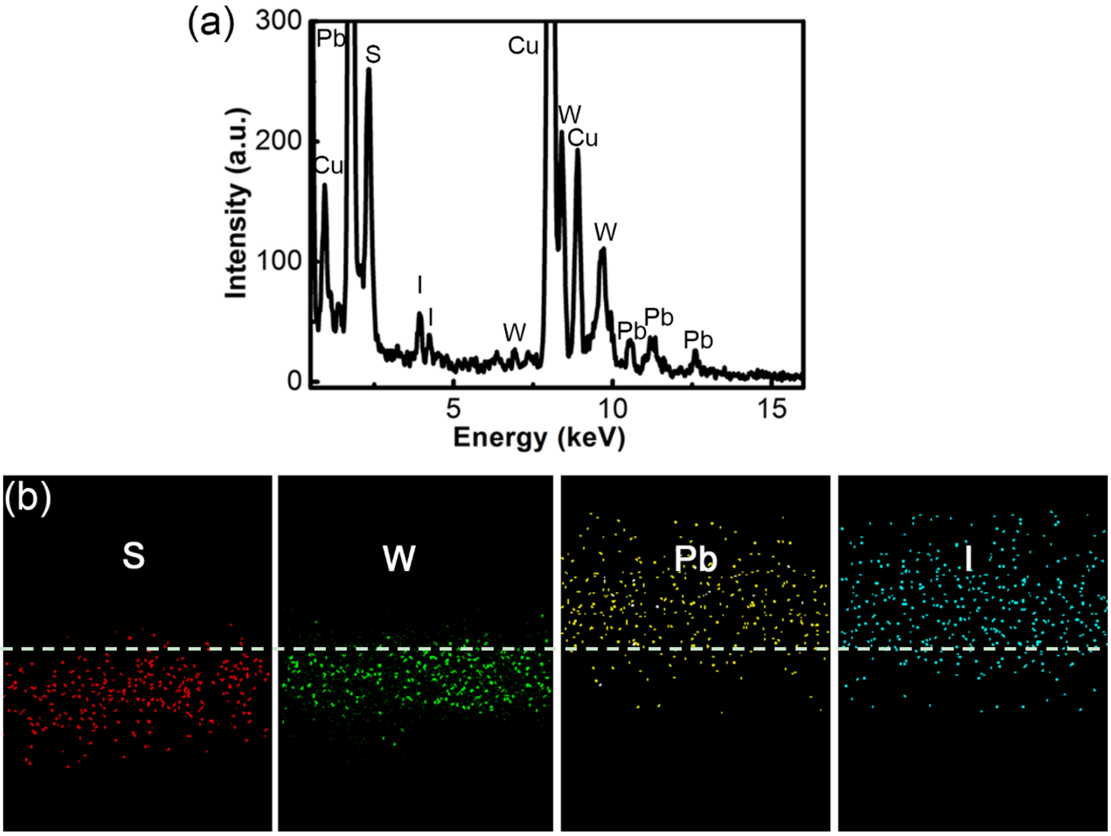

Figure S2. Corresponding EDS results of PbI_2/WS_2 heterostructure. (b) EDS mapping of S, W, Pb and I of a selected area cross section of PbI_2/WS_2 heterostructure. White dash line guides the interface.

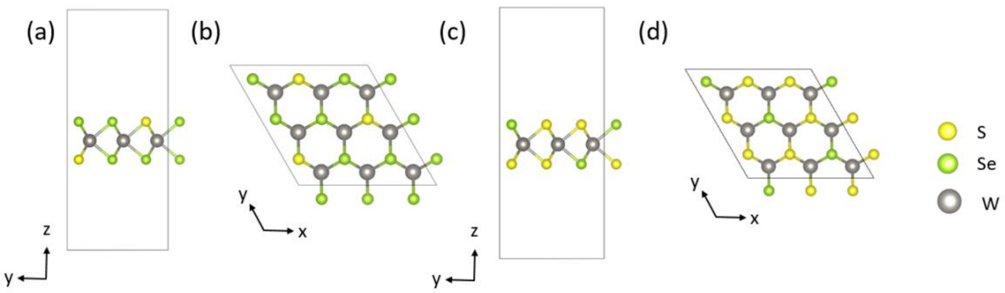

Figure S3. Lattice structure of $WS_{2(1\text{-}x)}Se_{2x}$. (a) Side view and (b) top view of the atomic structure of the 3×3 ×1 supercell monolayer $WS_{0.67}Se_{1.33}$. (c) Side view and (d) top view of the atomic structure of the 3×3 ×1 supercell monolayer $WS_{1.33}Se_{0.67}$.

REFERENCES

1. Duan, X.; Wang, C.; Fan, Z.; Hao, G.; Kou, L.; Halim, U.; Li, H.; Wu, X.; Wang, Y.; Jiang, J.; Pan, A.; Huang, Y.; Yu, R.; Duan, X., *Nano letters* **2016,** *16* (1), 264-9.
